# Unraveling the molecular landscape of lead-induced cochlear synaptopathy: a quantitative proteomics analysis

**DOI:** 10.3389/fncel.2024.1408208

**Published:** 2024-07-22

**Authors:** Pankaj Bhatia, Shomaila Mehmood, Nicole Doyon-Reale, Rita Rosati, Paul M. Stemmer, Samson Jamesdaniel

**Affiliations:** ^1^Institute of Environmental Health Sciences, Wayne State University, Detroit, MI, United States; ^2^Department of Family Medicine and Public Health Sciences, Wayne State University, Detroit, MI, United States

**Keywords:** cochlear synaptopathy, lead-induced ototoxicity, hearing loss, cochlear synaptosomes, synaptic vesicle cycle

## Abstract

**Introduction:**

Exposure to heavy metal lead can cause serious health effects such as developmental neurotoxicity in infants, cognitive impairment in children, and cardiovascular and nephrotoxic effects in adults. Hearing loss is one of the toxic effects induced by exposure to lead. Previous studies demonstrated that exposure to lead causes oxidative stress in the cochlea and disrupts ribbon synapses in the inner hair cells.

**Methods:**

This study investigated the underlying mechanism by evaluating the changes in the abundance of cochlear synaptosomal proteins that accompany lead-induced cochlear synaptopathy and hearing loss in mice. Young-adult CBA/J mice were given lead acetate in drinking water for 28 days.

**Results:**

Lead exposure significantly increased the hearing thresholds, particularly at the higher frequencies in both male and female mice, but it did not affect the activity of outer hair cells or induce hair cell loss. However, lead exposure decreased wave-I amplitude, suggesting lead-induced cochlear synaptopathy. In agreement, colocalization of pre- and post-synaptic markers indicated that lead exposure decreased the number of paired synapses in the basal turn of the cochlea. Proteomics analysis indicated that lead exposure increased the abundance of 352 synaptic proteins and decreased the abundance of 394 synaptic proteins in the cochlea. Bioinformatics analysis indicated that proteins that change in abundance are highly enriched in the synaptic vesicle cycle pathway.

**Discussion:**

Together, these results suggest that outer hair cells are not the primary target in lead-induced ototoxicity, that lead-induced cochlear synaptopathy is more pronounced in the basal turn of the cochlea, and that synaptic vesicle cycle signaling potentially plays a critical role in lead-induced cochlear synaptopathy.

## 1 Introduction

Environmental contamination with heavy metal lead is a growing concern because lead exposure affects human health, particularly that of children, and pregnant and lactating women, who are more vulnerable to its toxic effects (Choi et al., 2008; Tiwari et al., 2012; Olufemi et al., 2022). According to the World Health Organization, out of the 2 million deaths worldwide that are related to chemical exposure, nearly half of the deaths are linked to lead exposure (WHO, 2023a). Some of the common sources of lead exposure include mining, industrial pollutants, paints, contaminated foods, and water (Guidotti et al., 2007; Obeng-Gyasi, 2019; Kumar et al., 2020). Lead enters the body through multiple routes, accumulates over time, and causes severe health problems. Even at lower concentrations, lead can negatively impact health (Wani et al., 2015). The nervous system is particularly susceptible to the toxic effects of lead. Chronic exposure to lead is associated with an increased risk of neurodegenerative disorders, such as Alzheimer’s and Parkinson’s disease (Wang et al., 2020). The lead-induced neurotoxicity is characterized by disruptions in synaptic transmission, oxidative stress, and inflammation within the central nervous system (Hossain et al., 2016; Mahmoud and Sayed, 2016; Mohanraj et al., 2022). Workers exposed to inorganic lead experience reduced sensory and motor conduction velocities, indicating damage to the peripheral nervous system (Zheng et al., 2011). Even asymptomatic workers with chronic exposure to lead have subclinical damage to peripheral nerves (Seppäläinen and Hernberg, 1980; Bilińska et al., 2005).

In addition to the other well-known neurotoxic effects, lead exposure is linked to adverse auditory effects, including hearing loss (Park et al., 2010; Jamesdaniel et al., 2018; Zhang et al., 2019). Lead crosses the blood–brain barrier, injures the astrocytes in rat hippocampus, and potentially affects the central auditory pathway leading to auditory deficits (Selvín-Testa et al., 1991; Castellanos and Fuente, 2016; Shvachiy et al., 2023). Additionally, chronic lead exposure can damage the peripheral auditory pathway (Holdstein et al., 1986; Zou et al., 2003), because it adversely impacts axonal integrity and myelin organization (Brubaker et al., 2009), which can result in reduced nerve conduction velocity and impaired signal transmission efficiency (Takeuchi et al., 2021; Aktürk et al., 2022). Chronic exposure to lead for 60 days significantly reduced the count of spiral ganglion neurons, delayed the latency of auditory brainstem response (ABR) wave I, and caused hearing loss (Zhang et al., 2019). Lead exposure is also reported to modify neuronal structural proteins, increase phosphorylation of neurofilament proteins in auditory brainstem nuclei, cause neuritic beading in axons, impair axonal transport, and affect brainstem conduction time and temporal auditory processing (Jones et al., 2008). However, the molecular mechanism by which lead exposure causes auditory dysfunction is not fully understood.

Oxidative stress appears to play a central role in mediating the ototoxic effects of lead. Lead exposure in rats triggered oxidative stress within the cochlea, leading to degenerative changes in spiral ganglion neurons and subsequent hearing loss (Xue-wen et al., 2011; Hu et al., 2019). Chronic lead exposure also downregulated several cochlear genes that encode critical antioxidant enzymes (Jamesdaniel et al., 2018). Another recent study showed that lead exposure increased nitrative stress in the spiral ganglion cells and disrupted the ribbon synapses resulting in cochlear synaptopathy (Rosati et al., 2022). To gain further insights into the molecular landscape of lead-induced cochlear synaptopathy, the current study investigated the lead-induced changes in cochlear synaptosomal proteins by high throughput proteomics analyses. Cochlear synaptosomal proteins extracted from lead-exposed mice were analyzed by tandem mass spectrometry and critical targets and molecular pathways associated with lead-induced cochlear synaptopathy were identified by bioinformatics analyses.

## 2 Experimental procedures

### 2.1 Experimental design and statistical rationale

**Figure 1:**
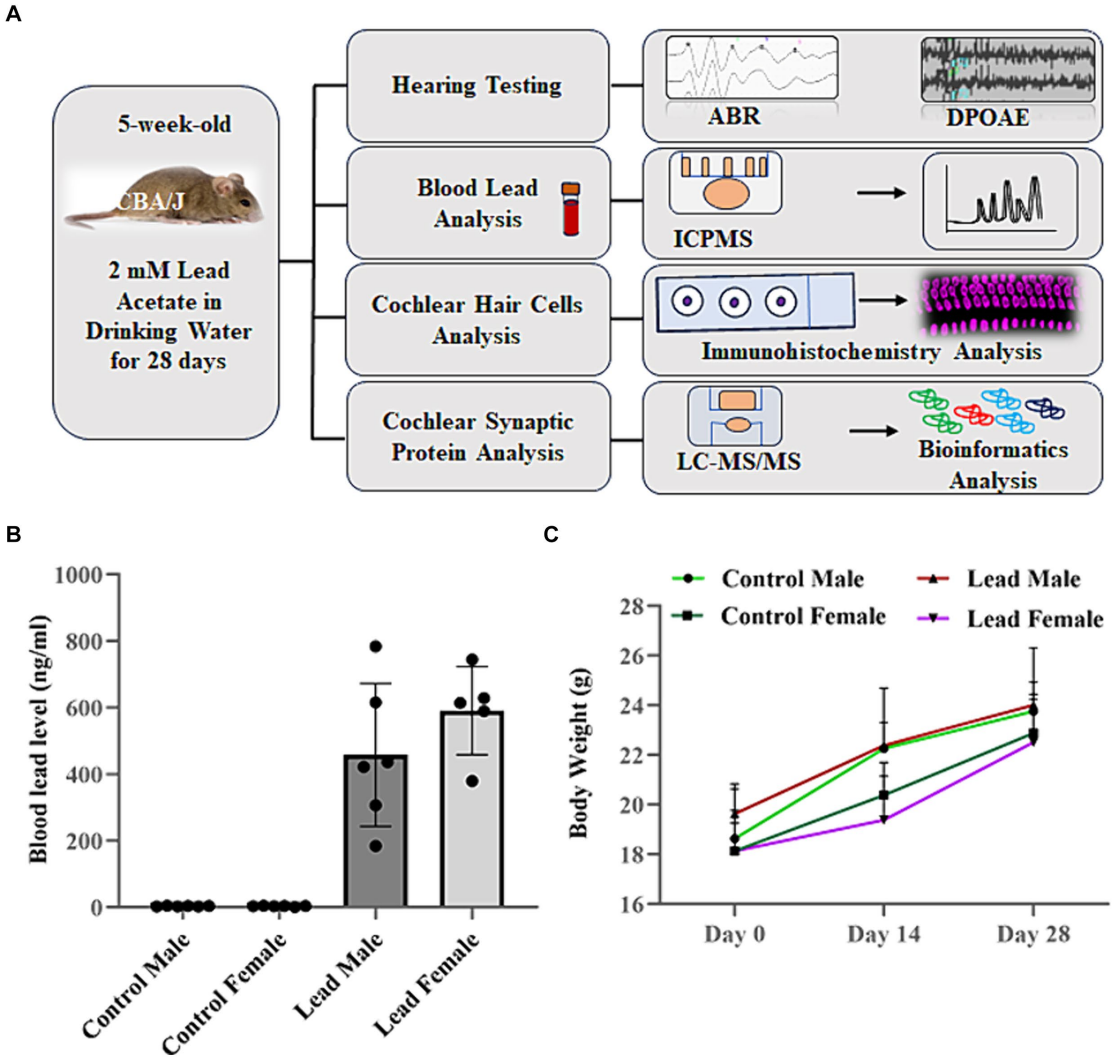
Overview of research design. **(A)** CBA/J mice were exposed to lead in drinking water for 28 days. Lead body burden was analyzed by assessing the blood lead levels 1 day after the last exposure. Hearing loss was assessed by measuring the shift in ABR thresholds while the outer hair cell activity was assessed by measuring DPOAE amplitudes. Hair cell loss and ribbon synaptic disruption was assessed by immunohistochemical analysis of cochlear sensory epithelium. The lead-induced changes in abundance of synaptosomal proteins and associated signaling pathways were assessed by LC–MS/MS and bioinformatics analysis, respectively. **(B)** ICPMS analysis indicated that lead exposure increased the blood lead levels in male and female mice (457.60 ± 87.8 ng/mL in males and 590.72 ± 59.28 ng/mL in females). Animals in the control groups consumed normal water, while those in the lead groups consumed water containing 2 mM lead acetate for 28 days. The results are expressed as mean ± standard error mean, *n* = 5–6. **(C)** Measurement of body weight on day 0, 14, and day 28 indicated that exposure to lead did not alter the normal weight gain of CBA/J mice. The results are expressed as mean ± standard deviation, *n* = 6. ABR, auditory brainstem response; DPOAE, distortion product otoacoustic emissions; ICPMS, inductively coupled plasma mass spectrometry; LC–MS/MS, liquid chromatography tandem mass spectrometry.

Auditory deficits and proteomics profile of cochlear synaptosomes were analyzed in mice exposed to lead (Figure 1A). The electrophysiology experiments were done in four to eight biological replicates, immunohistochemistry and immunoblotting experiments were done in a minimum of three biological replicates, and the mass spectrometry experiments were conducted using four to six biological replicates in each group.

### 2.2 Animals

Five-week-old male and female CBA/J mice (Jackson Laboratories, Bar Harbor, ME, United States) were used in this study. The animals were provided with food and water *ad libitum* and maintained in accordance with the standard guidelines of the National Institutes of Health. The University Institutional Animal Care and Use Committee reviewed and approved the experimental protocol (IACUC-22-01-4298).

### 2.3 Lead exposure

After baseline hearing tests, mice with normal hearing were randomly divided into two sets. Control mice were provided regular drinking water over a 28-day period, while the mice in the lead exposure group were provided with drinking water containing lead acetate (2 mM; Cat # 316512, Sigma-Aldrich, St. Louis, MO, USA) for 28 days. After the post-exposure hearing tests on the 29th day, the animals were euthanized, and blood and cochlea samples were collected. The animals’ appearance and behavior were monitored throughout the study, and their body weight was measured every other week.

### 2.4 Inductively coupled plasma mass spectrometry

To analyze the blood lead levels, 75-μl of blood was mixed with an equal volume of 0.1% Triton X-100 and then diluted with 300 mL of 2% nitric acid. The mixture was incubated for 1–2 h and then centrifuged. The sample was diluted again with 2% nitric acid for a final 50-fold dilution. A standard curve was generated using lead concentrations ranging from 0.05 to 200 ppb. The analysis was conducted using an Agilent 7,700X Series inductively coupled plasma mass spectrometer (ICP-MS).

### 2.5 Auditory brainstem responses and distortion product otoacoustic emissions

Auditory brainstem responses (ABR) and distortion product otoacoustic emissions (DPOAE) were recorded using Tucker Davis Technologies (TDT) System III hardware. The BioSigRZ software (v5.7.6.) was used to present sound stimuli. Mice were anesthetized with isoflurane (4% induction, 1.5% maintenance with 1 L/min O_2_) and placed on a temperature-controlled heating pad maintained at 38°C inside the soundproof chamber (ECKEL-AB-4230, Morrisburg, Ontario). For recording ABRs, the electrodes were placed subcutaneously at the vertex of the skull (active), mastoid region under the left ear (reference), and the right ear (ground). The sound stimuli of clicks and tone bursts at frequencies of 4, 8, 16, 24, or 32 kHz were delivered into the external auditory meatus. The ABR waveforms were obtained by averaging 512 stimulus presentations, delivered at 21/s. The sound intensity varied in 5-dB intervals starting from 90 dB Sound Pressure Level (SPL). The hearing threshold was determined by identifying the lowest intensity of stimuli that elicited Wave I peak. The amplitude (difference between the voltage levels of the peak and the trough) and the latency (the time elapsed from the onset of the stimulus to the peak of the first wave) of Wave I were measured using 90-dB SPL stimuli.

DPOAEs were measured using two primary tones, f1 and f2, at an f2/f1 ratio of 1.2. The L2–L1 was held at +10 dB for L1 levels ranging from 80 dB SPL to 20 dB SPL in 10 dB increments. The Tucker-Davis Technology (TDT) RZ6 system was used to generate the stimulus, while and f1 and f2 were delivered by using multifield magnetic speakers (TDT, Alachua, FL, United States). The frequency of f2 varied from 4 kHz to 40 kHz. SPLs at the cubic difference frequency (2f1–f2) were measured using the ER10B + probe microphone (Etymotic Research, Inc., Elk Grove Village, IL, United States), along with hardware and software from TDT. Distortion product data were collected every 20.971 ms and averaged 512 times while a 100-kHz band surrounding 2f1–f2 was used for measuring the noise floor.

### 2.6 Immunohistochemistry of cochlear surface preparations

After euthanizing the mice, the temporal bones were quickly extracted on ice-cold PBS, the blood in the cochlea was flushed out by perfusing with 4% paraformaldehyde in PBS (7.4 pH) through the oval window, and the cochlear tissue was fixed for 24 h. Then the cochlea was decalcified in 100 mM ethylenediaminetetraacetic acid disodium salt (EDTA) solution in PBS (7.4 pH) for 48–72 h. Fully decalcified tissue was micro-dissected in ice-cold PBS solution under a stereomicroscope (Olympus model # SZH-ILLK) and the cochlear sensory epithelium was separated from the bony capsule and the lateral wall. Then the sensory epithelium was cut into apex, middle, and basal sections.

For the immunolabeling, each cochlear section was first incubated in a blocking solution containing 10% normal goat serum for 2 h at room temperature. Then the tissue was incubated with primary antibodies overnight and then with secondary antibodies for 2 h at 4°C. The primary antibodies used in this study were: rabbit anti-glutamate receptor 2 (GluR2), clone 6C4 (1:200, IgG, Millipore # MAB397), mouse anti-C-terminal-binding protein-2 (CtBP2) (1:400, IgG1, BD Biosciences # 612044), and mouse anti-myosin VIIa monoclonal antibody (1:500, IgG2a, Santa Cruz biotechnology # 74516). The secondary antibodies used were goat anti-rabbit Alexa Fluor (AF) 647 (1:500, IgG, Thermo Fisher Scientific # 21244), goat anti-mouse AF 568 (1:500, IgG1, Thermo Fisher Scientific # 21124), and goat anti-mouse AF 488 (1:500, IgG2a, Thermofisher Scientific # 21131). The ProLong Gold antifade with DAPI was used for mounting the tissue, and a coverslip was secured from the side using Biotium CoverGrip Coverslip Sealant (Fisher Scientific # NC0154994).

### 2.7 Confocal microscopy and image processing

All images were captured using a Zeiss confocal microscope LSM 800 and analyzed using Zeiss Zen Blue 3.7 and ImageJ/Fiji software (v 1.46r). Immunolabeling of OHCs and IHCs were captured using 40x plan apochromatic objective and 1.3 numerical aperture. The immunolabeling of presynaptic ribbons (CtBP2) and postsynaptic ribbons (GluR2) were captured using a 63x-magnification objective lens with an effective numerical aperture of 1.4. Z-stacks of 10–15 slices for hair cells and 20–25 slices for CtBP2 and GluR2 puncta were compressed to make maximum intensity projection. The localization of CtBP2 and GluR2 puncta were examined to assess the pairing of the synapses using Zen software (v 3.7). Hair cell counts were quantified in 100 μm segments of the apex (4–8 kHz), middle (16–24 kHz), and basal (32 kHz) regions of the cochlea, and the ribbon synapses were counted in 6-IHCs and averaged to get ribbon synapses/IHC. The number of paired synaptic ribbons was counted using the 3D Object Counter of ImageJ software.

### 2.8 Cochlear synaptosomal protein extraction

Cochlear tissue was dissected and the synaptosomal proteins were extracted from the entire cochlea by homogenizing the tissue in Syn-PER reagent (Thermo Scientific # 87793, Rockford, IL, USA)following the manufacturer’s protocol. Briefly, the homogenate was centrifuged at 1200 × *g* for 10 min at 4°C, and the supernatant was transferred to a new tube and centrifuged at 15,000 × *g* for 20 min at 4°C. Then, the supernatant was discarded, and the synaptosome pellets were resuspended in Syn-PER reagent. This step was repeated, and the supernatant was carefully discarded to obtain the pellets containing synaptosome proteins.

### 2.9 Immunoblotting

The enrichment of the synaptosomal proteins was verified by immunoblotting with anti-SNAP-25. A total of 5 μg of proteins from the synaptosomal fraction, non-synaptosomal fraction (supernatant), and the total cochlear homogenate were separated on 4–20% gradient SDS-Page gels, transferred to PVDF membranes, and probed with goat polyclonal SNAP-25 antibody (1:500, IgG, ThermoFisher Scientific # PA1-9102, Waltham, MA, USA). The bands were developed using a chemiluminescence detection reagent (Thermo Fisher Scientific # 34076), and the bands were visualized using FluorChem E System (ProteinSimple, San Jose, CA, USA). Mouse monoclonal β-Actin Antibody (1:500, IgG1κ, Santa Cruz Biotechnology # sc-47778, CA, USA) was used for normalization.

### 2.10 Liquid chromatography tandem mass spectrometry

Synaptosomal pellets were resuspended in water and then combined with an equivalent volume of 2.5% Lithium Dodecyl Sulfate (Sigma L9781, St. Louis, MO, USA) in 40 mM Triethylammonium bicarbonate buffer (TEAB, Honeywell Fluka, 60–044-974, Suwanee, GA, USA). The samples were homogenized in a Bullet Blender (Next Advance, BT12LT, Baltimore, MD, USA) utilizing a single 3.2 mm stainless steel bead in each tube (Next Advance, SSB32, Baltimore, MD, USA) for 1 min, incubated for 5 min at 95°C to deactivate enzymes then filtered through 0.8 mL Pierce spin columns to decrease the viscosity. Samples were reduced by adding 5 mM DL-Dithiothreitol (DTT, Sigma, D5545, St. Louis, MO, USA) and incubated for 30 min at 37°C. Then the samples were alkylated by adding 15 mM Iodoacetamide (IAA) (Sigma, I1149, St. Louis, MO, USA) and further incubated for 30 min at room temperature in the dark. The alkylation process was halted by adding 5 mM DTT, the samples were acidified by adding 10% of the initial volume of 12% phosphoric acid and precipitated by addition of 5 volumes of methanol (MeOH) and incubating overnight at −20°C. Finally, the samples were centrifuged at 4,000 × *g* for 5 min to separate the pellets, which were then washed with 80% MeOH and 1% TEAB, and the washed pellets were air-dried and resuspended in 50 μL of 40 mM TEAB. Digestion was achieved using trypsin (Promega) at a ratio of 1:25 (w/w), and the samples were incubated at 47°C for 1 h, followed by 3 h at 37°C. The resultant peptides were solubilized in 20 μL of 0.1% formic acid (FA), and 10 μL of this solution was utilized for proteomics analysis. Final analysis was performed on the samples using a Thermo Scientific Vanquish-Neo chromatography system with an Acclaim PepMap 100 trap column (100 μm × 2 cm, C18, 5 μm, 100 Å), and Thermo Scientific Easy-Spray PepMap RSLC C18 75 um x25 cm column. A gradient starting at 4% acetonitrile and finishing at 30% acetonitrile 100 min later was used for all samples. LC–MS/MS with Data Independent Acquisition (DIA) was performed on an Orbitrap Eclipse MS system. MS1 spectra were acquired at 120,000 resolutions in the 400 to 1,600 Da mass range with an AGC of 1e6. MS2 spectra were acquired using variable windows with a resolution set at 15,000.

### 2.11 Bioinformatics analysis

Spectronaut-18 software (Biognosys; v18) was used to quantitatively profile the proteomic changes and identify the differentially abundant synaptosomal proteins. A fold change of ±1.5 was chosen as a criterion for selecting proteins of interest. Official gene symbols of high and low-abundant proteins were used for GO annotation and KEGG (Kyoto Encyclopedia of Genes and Genomes) pathway enrichment analysis. Shiny GO v0.80 bioinformatics tool (Ge et al., 2020) was used with FDR = 0.05, to identify the signaling pathways regulated by genes of differentially expressed proteins. The top 30 biological processes (BP), and KEGG pathways associated with lead-induced changes in the abundance of cochlear synaptosomal proteins were identified by the bioinformatics analysis. The Search Tool for the Retrieval of Interacting Genes (STRING) database Version 12.0 was used for protein–protein interaction (PPI) analysis (Szklarczyk et al., 2016).

### 2.12 Statistical analysis

Data were analyzed using Microsoft Excel’s Data Analysis Toolkit (Office Professional Plus 2016, Microsoft, Redmond, WA, USA) and GraphPad Prism (v10, La Jolla, CA, USA). Volcano plots were generated using Spectronaut software. Statistical analysis employed a two-tailed unpaired t-test, with significance set at *p* < 0.05. The outcomes are presented as mean ± standard deviation or standard error mean.

## 3 Results

### 3.1 Lead exposure via drinking water increased blood lead levels

Exposure of mice to drinking water containing 2 mM lead acetate for 28 days significantly increased the blood lead levels. Figure 1B shows the average blood lead levels in control and lead-exposed mice. The blood lead load in lead-exposed male and female mice was 457.60 ± 87.78 ng/mL and 495.68 ± 106.66 ng/mL, respectively, and was significantly higher than the blood lead levels detected in control male (2.78 ± 0.36 ng/mL) and female (2.54 ± 0.55 ng/mL) mice. This indicated that the 28-day exposure used in this study increased the lead body burden in mice. However, this lead exposure did not alter the body weight of the animals (Figure 1C).

### 3.2 Hearing thresholds are altered by lead exposure

**Figure 2:**
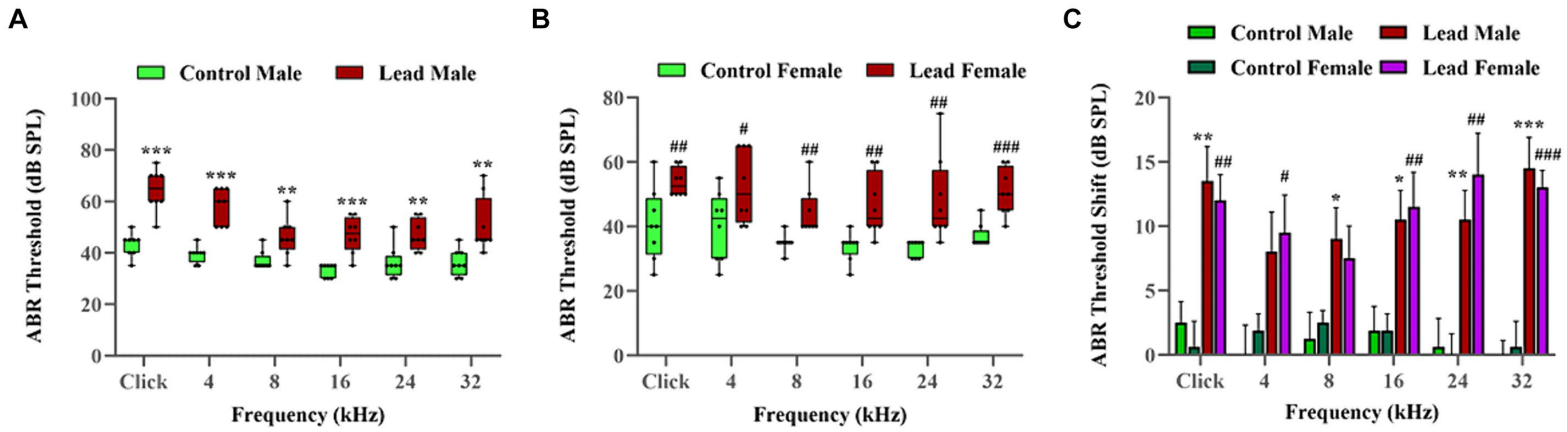
Lead-induced changes in the hearing thresholds. **(A,B)** Lead exposure elevated the ABR thresholds in both male and female mice, respectively. The hearing thresholds recorded using the left ear are illustrated. The results are expressed as mean ± standard error mean; *n* = 8. **(C)** The ABRs indicated that lead exposure induced a significant shift in the hearing thresholds (ranging from 8.00 to 14.50 dB in male mice and 7.50 to 14.00 dB in female mice) across multiple frequencies. Further analysis using *t*-tests indicate that the lead-induced threshold shifts at high frequencies (32 kHz) are significant (*p* < 0.05) compared to those at lower frequencies (8 kHz). The ABR threshold shifts represent the difference in thresholds measured at a young age (5 weeks) and 4 weeks later. The results are expressed as mean ± standard error mean; *n* = 8–10. * Indicates *p* < 0.05, **p < 0.01, and ***p < 0.001 vs. control male; # indicates *p* < 0.05, ##*p* < 0.01, and ###*p* < 0.001 vs. control female. ABR, auditory brainstem response.

ABRs were recorded to evaluate the effect of lead exposure on hearing thresholds. ABRs indicated that while the control mice had normal hearing across all frequencies, lead exposure significantly increased the hearing thresholds across multiple frequencies. In males, the hearing thresholds were elevated in ABRs elicited with pure-tone stimuli at 8, 16, 24, and 32 kHz, and click, while in females, the hearing thresholds were elevated in ABRs elicited with pure-tone stimuli at 4, 16, 24, and 32 kHz, and click. These results indicated that lead exposure for 28 days induced significant hearing loss in mice. The lead-induced shift in hearing thresholds was more pronounced in ABRs elicited with pure-tone stimuli at higher frequencies (24 and 32 kHz) and click, and the shifts were similar in both male and female mice. Although a two-tailed parametric *t*-test showed a significant difference in the shifts between 8 and 32 kHz (*p* = 0.0318), suggesting that the higher frequency regions may be more vulnerable to lead-induced ototoxicity, the ANOVA test did not show a significant difference. The hearing threshold shifts also indicated that both sexes are equally susceptible to lead-induced hearing impairment (Figures 2A–C). As we did not record the ABR a few days or weeks after the cessation of lead treatment, we do not have the data to show if the hearing loss will recover.

### 3.3 Outer hair cells are not affected by lead exposure

**Figure 3:**
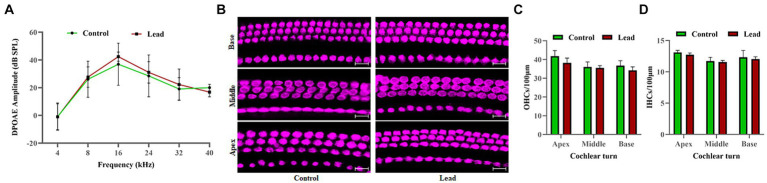
Effect of lead exposure on the hair cells. **(A)** The DPOAEs recorded using 4, 8, 16, 24, 32, and 40 kHz f2 stimuli indicated that the DPOAE amplitudes elicited using 70 dB SPL (L1) stimuli were similar in both control and lead exposed animals. The results are expressed as mean ± standard deviation; *n* = 4–8. **(B)** Immunohistochemical analysis with anti-myosin VIIa indicated that lead exposure did not affect the viability of both the OHCs and IHCs in the apex, middle, and basal regions of the organ of Corti. **(C,D)** OHCs and IHCs counts were carried out, and the mean number of cells per 100 μm section of the apex, middle, and basal region of the cochlea were displayed. Images are representative of three biological replicates. Scale bar = 20 μm. DPOAE, distortion product otoacoustic emissions; OHCs, outer hair cells; IHCs, inner hair cells.

DPOAEs were recorded to evaluate the effect of lead exposure on OHC activity. The DP amplitudes measured in both male and female mice with f2 stimuli at 4, 8, 16, 24, 32, and 40 kHz indicated that the DPOAEs were similar in both control and lead-exposed mice (Figure 3A). This suggested that the OHC activity is not affected by lead exposure. Additionally, the effect of lead exposure on hair cell viability was assessed by immunohistochemistry analysis of cochlear surface preparations. Evaluation of myosin-VIIa immunostaining indicated that there was no hair cell loss in the apical, middle, and basal regions of the cochlea in both control and lead-exposed mice (Figure 3B). This suggested that lead exposure did not affect the viability of hair cells in the cochlea. Furthermore, the quantification of OHCs and IHCs confirmed no loss of hair cells in the apex, middle, and basal regions of the cochlea after lead exposure (Figures 3C,D).

### 3.4 Cochlear synaptic transmission is affected after lead exposure

**Figure 4:**
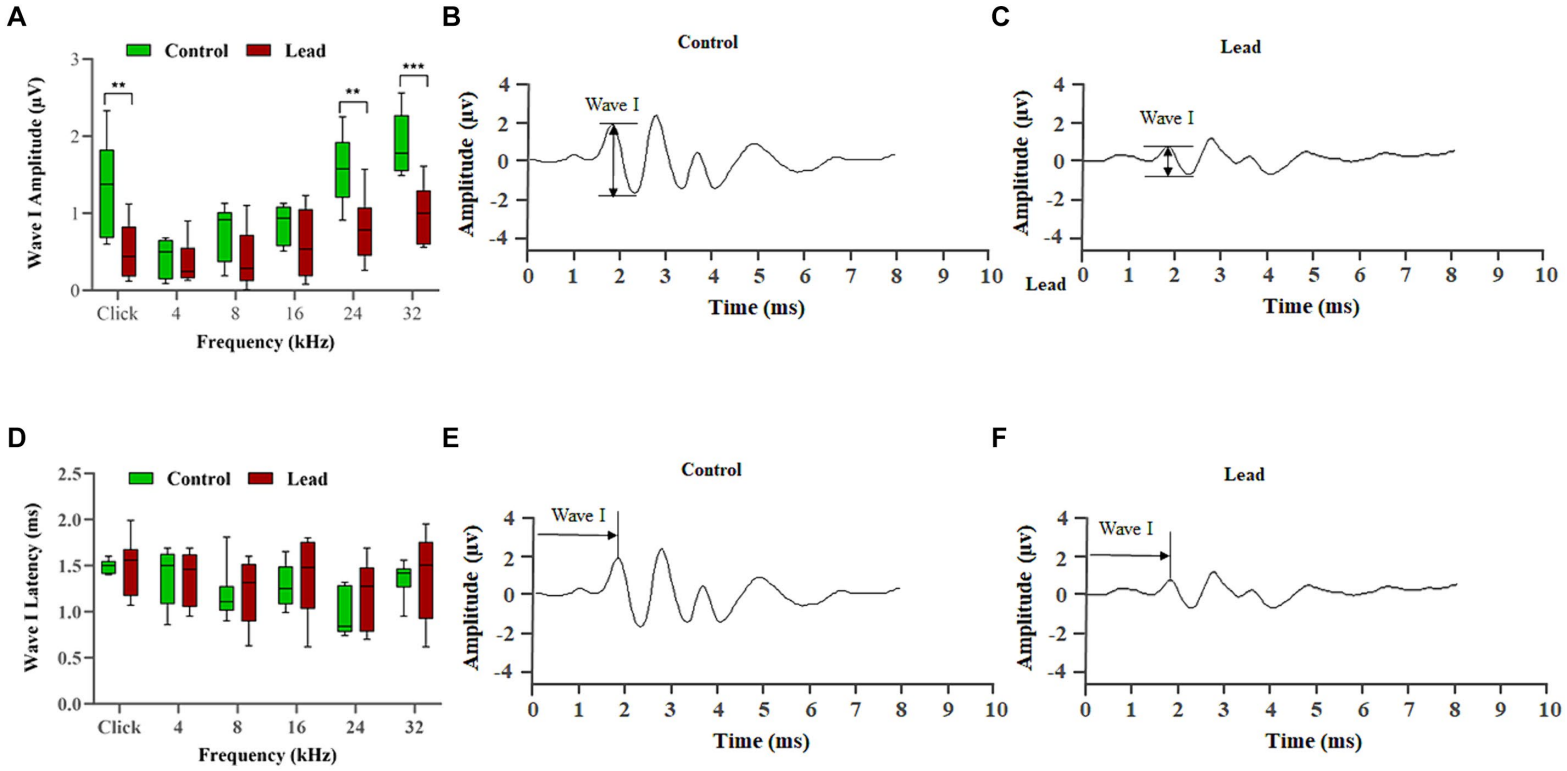
Effect of lead on the signal transmission in cochlear nerve fibers. **(A–C)** Lead exposure significantly reduced the amplitude of ABR wave I elicited using click and 24, and 32 kHz stimulus frequencies suggesting a decrease in the number of neurons firing in response to a stimulus. The lead-induced changes were greater at the higher frequencies, indicating that the basal region is more susceptible to lead-induced toxicity. The results are expressed as mean ± standard error mean; *n* = 8. ** Indicates *p* < 0.01, and ***p < 0.001 vs. control male. **(D–F)** Lead exposure did not alter the latency of ABR wave I, which suggests that the speed of neural conduction is not affected. Data collected from the left ear is illustrated. The results are expressed as mean ± standard error mean; *n* = 8. ABR, auditory brainstem response.

The amplitude and latency of ABR wave-I were evaluated to assess the effect of lead exposure on cochlear synaptic transmission. Measurement of ABR wave-I amplitude recorded with pure-tone and click stimuli at 90 dB SPL indicated that lead exposure induced a significant reduction in wave-I amplitudes across multiple frequencies (24, and 32 kHz) when compared to the control group (Figures 4A–C). The decrease in wave-I amplitude was more pronounced at higher frequencies (24 and 32 kHz) than at lower frequencies (4, 8, and 16 kHz). However, the ABR wave-I latency was similar in both the control and lead-exposed groups, suggesting that wave-I latency is not affected by the 28-day lead exposure (Figures 4D–F). Together, these results suggest that the lead exposure paradigm used in this study decreases the number of firing neurons within the cochlea and thereby affects the cochlear synaptic transmission.

### 3.5 Lead exposure disrupted the pairing of cochlear ribbon synapses

**Figure 5:**
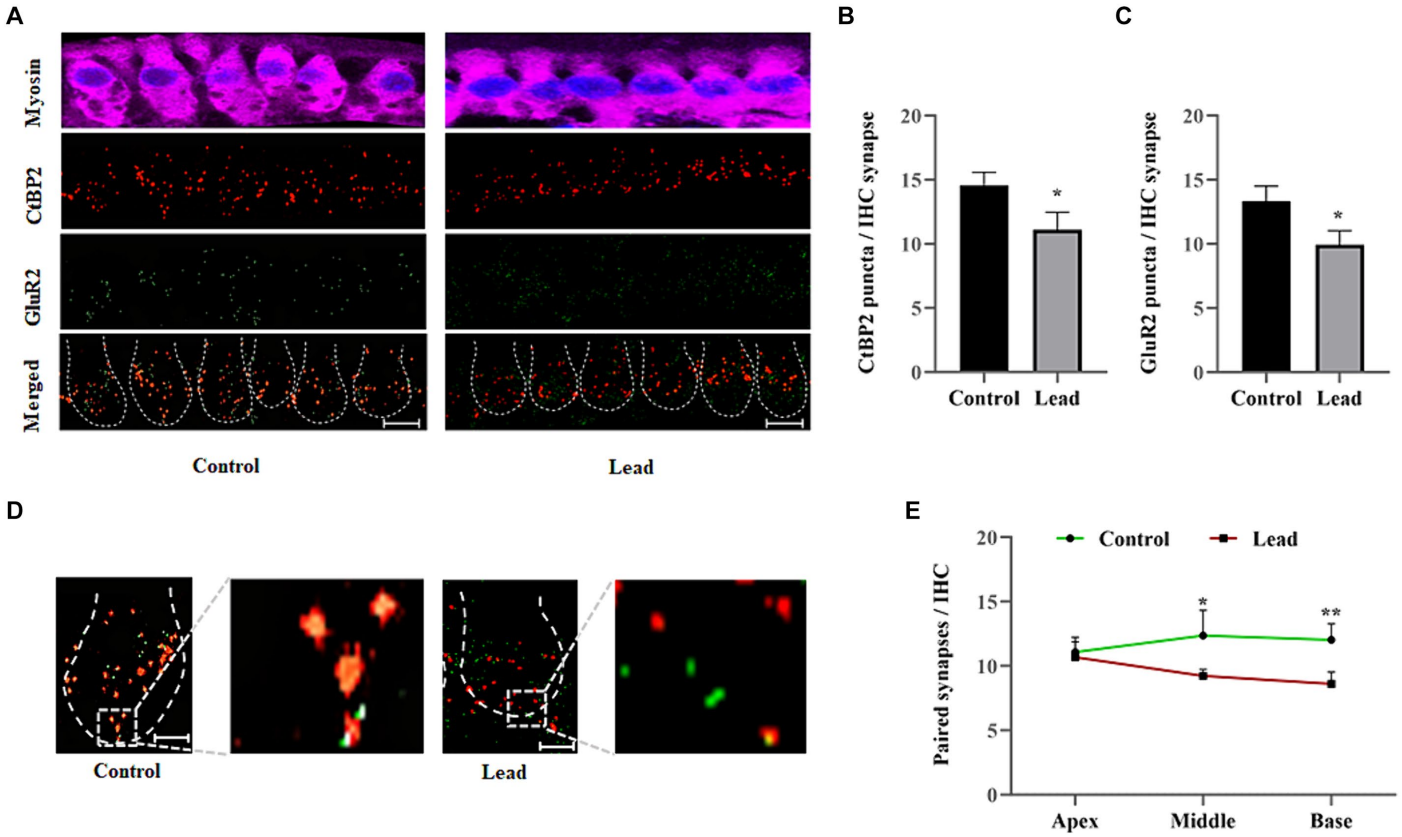
Lead-induced changes in the cochlear ribbon synapses. **(A)** Immunohistochemical staining of ribbon synapses in the basal turn of the cochlea indicated that lead exposure affected the presynaptic ribbons and postsynaptic receptors. The presynaptic ribbons were stained with anti-CtBP2 (red) and postsynaptic receptors were stained with anti-GluR2 (green). The images are representative of three biological replicates, scale bar = 10 μm. **(B,C)** Quantification of the CtBP2 and GluR2 puncta per inner hair cell synapse indicated that lead exposure significantly decreased the number of puncta. The data represent the basal turn of the organ of Corti. The results are expressed as mean ± standard deviation; *n* = 3. * Indicates *p* < 0.05 vs. control. **(D)** The pairing of the presynaptic ribbons with postsynaptic receptors was examined by documenting the yellow stained puncta (clearly visible in the zoomed image), which indicated paired ribbon synapses. The images are representative of three biological replicates, scale bar = 10 μm. **(E)** Quantification of the paired synapses per IHC indicated a significant decrease in the basal cochlear turn after lead exposure. The results are expressed as mean ± standard deviation; *n* = 3–6. * Indicates *p* < 0.05 and **p < 0.01 vs. control. CtBP2, C-terminal-binding protein-2; GluR2, glutamate receptor 2; IHC, inner hair cell.

The cochlear ribbon synapses were evaluated by immunohistochemistry analysis of presynaptic ribbon (CtBP2) and postsynaptic receptor (GluR2) markers to determine the effect of lead exposure on the pairing of synapses. Lead exposure decreased the number of CtBP2 and GluR2 puncta located at the presynaptic and postsynaptic sides of the IHC synapse, respectively (Figures 5A–C). The absence of CtBP2 staining in the nuclei of IHCs in our images is probably due to the orientation of the tissue on the slide and the variations in the focal plane that were specifically used to capture high-quality images of ribbon synapse. The localization of the CtBP2 and GluR2 puncta was examined to quantify the number of paired synapses (Figure 5D), which indicated that the paired synapse count was significantly decreased in the middle and basal turn of the cochlea in lead-exposed mice (Figure 5E). Further, the lead-induced decrease in pair synaptic count was more pronounced in the basal region of the cochlea. These results suggest that the lead-induced reduction in the number of paired synapses contributes to the hearing impairment observed after lead exposure. Additionally, the basal region appears to be more susceptible to lead-induced ototoxicity.

### 3.6 Lead exposure altered the abundance of cochlear synaptosomal proteins

**Figure 6:**
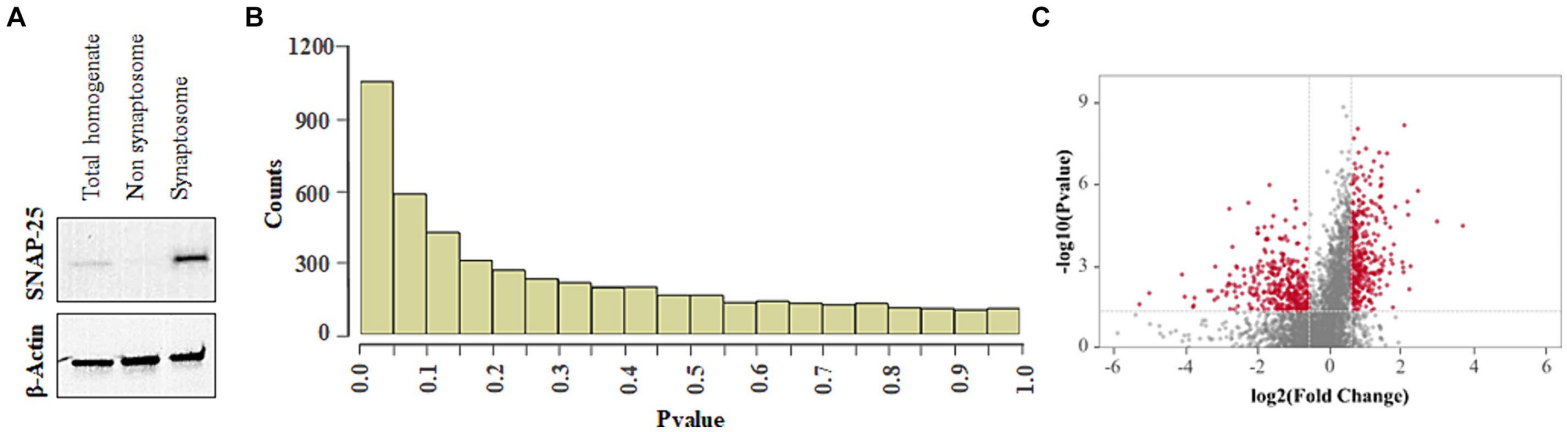
Effect of lead on cochlear synaptosomal proteins. **(A)** Immunoblot indicates the enrichment of SNAP-25 in the synaptosomal fraction. The images are representative of three biological replicates. **(B)** *p*-value histogram of lead-induced changes in the abundance of cochlear synaptosomal proteins indicates an anti-conservative graph where the data follows the null distribution. **(C)** Volcano plot illustrates the fold changes of 4,947 cochlear synaptosomal proteins identified using mass spectrometry analysis. The plot indicates that lead exposure increased the abundance of 352 synaptic proteins (red dots on the right side) and decreased the abundance of 394 synaptic proteins (red dots on the left side). A Fold change of ±1.5 was used as a criterion for selecting high and low-abundant proteins. The horizontal red line represents *p* < 0.05. Results are expressed as the mean of four biological replicates.

The changes in the abundance of cochlear synaptosomal proteins were evaluated to identify potential targets of lead-induced disruption of cochlear ribbon synapses and cochlear synaptic transmission. Immunoblotting with SNAP25 indicated the enrichment of synaptosomal proteins in the analyzed samples (Figure 6A). Although we have verified the enrichment of some other synaptosomal proteins in our samples using western blots in previous studies also (Shahab et al., 2024), we did not verify that all the proteins detected in the mass spectrometry analysis are from the synaptosomal compartment. Analysis of the mass spectrometry data using Spectronaut software identified a total of 4,947 proteins. The *p*-value histogram of the identified proteins indicated an anti-conservative graph in which the data followed the null distribution (Figure 6B). Further analyses of the data applying the criteria of *p* < 0.05 and fold change ±1.5, identified 746 differentially abundant proteins. Of these, the abundance of 352 proteins was increased while the abundance of 394 proteins was decreased after lead exposure (Figure 6C).

### 3.7 Synaptic vesicle cycle machinery is affected in lead-induced cochlear synaptopathy

**Figure 7:**
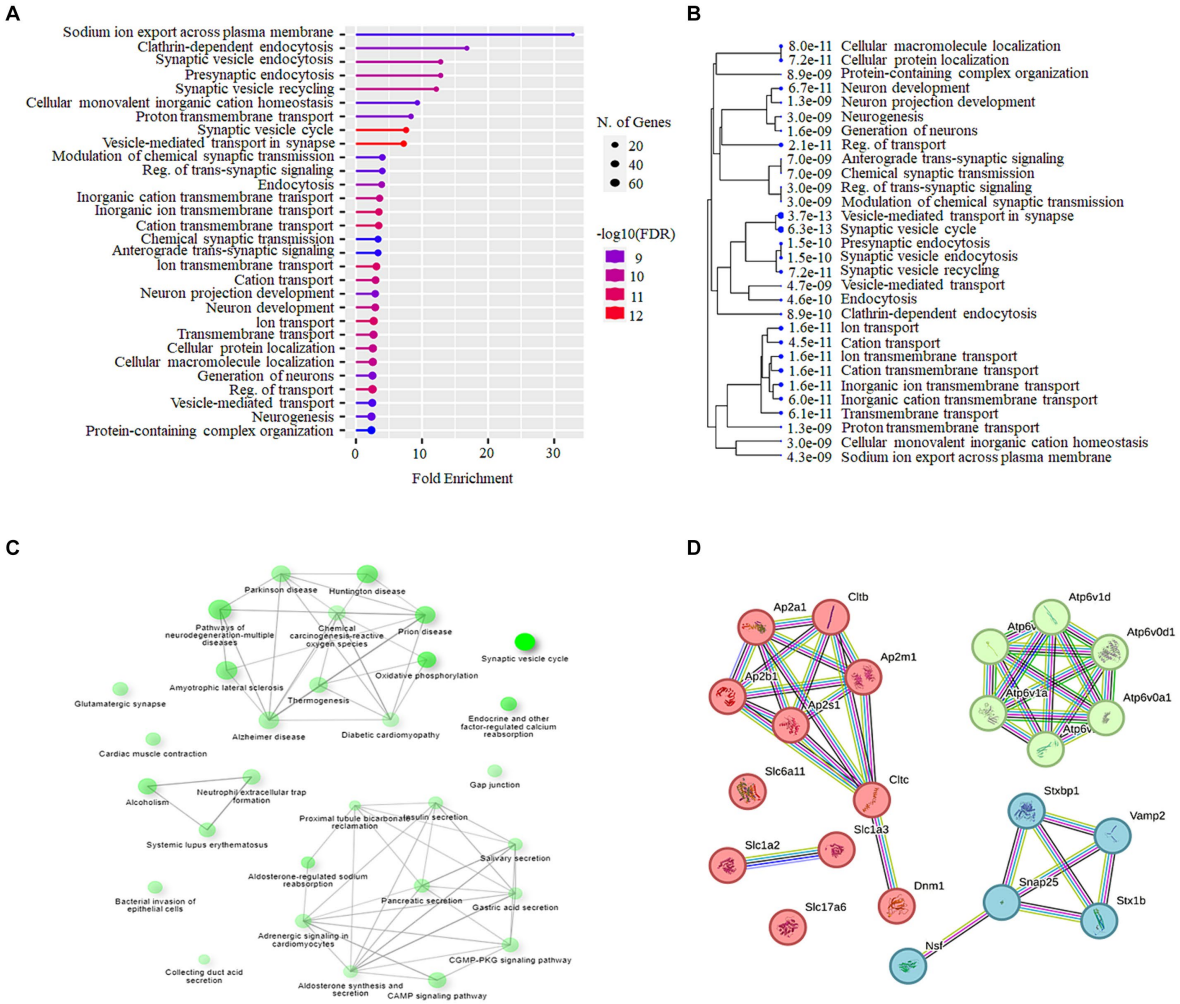
Bioinformatics analysis of cochlear synaptosomal proteins whose abundance was increased after lead exposure. **(A)** The GO analysis using the Shiny GO platform revealed the BPs enriched in the data set. The dot plot illustrates the fold enrichment, FDR, and the total number of proteins associated with these BPs. **(B)** The hierarchical clustering tree illustrates the clustering of significantly enriched BPs (*p*-value cutoff = 0.05). **(C)** The network analysis of enriched KEGG pathways shows the number of proteins in the pathway (indicated by the size of the node) and the percentage of overlapping proteins (indicated by the thickness of the edges). **(D)** The STRING interaction analysis of proteins associated with the synaptic vesicle cycle pathway illustrates the type of interaction with the highest confidence (0.900). Three main clusters, cluster 1 represented by red nodes, cluster 2 represented by green nodes and cluster 3 represented by light blue nodes were detected. GO, Gene Ontology; BPs, Biological processes; FDR, False discovery rate; STRING, Search Tool for the Retrieval of Interacting Genes.

**Figure 8:**
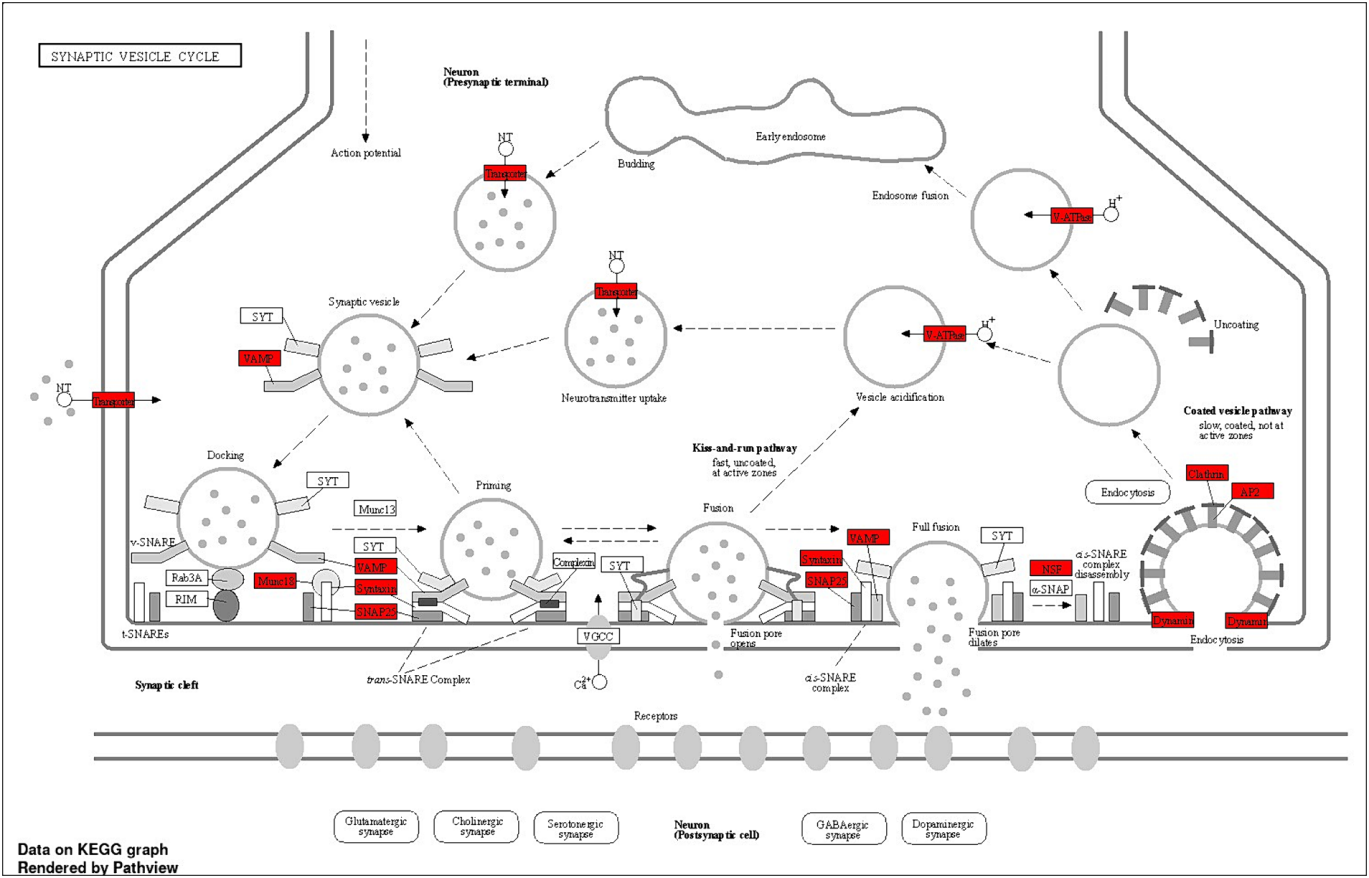
Schematic summary of the synaptic vesicle cycle pathway, illustrating key steps and proteins involved, including differentially expressed proteins. The dotted lines indicate the synaptic vesicle cycle, from neurotransmitter filling to release, followed by endocytosis. Budding: Neurotransmitters are actively transported into the synaptic vesicle, initiating the filling process. Docking: Vesicles filled with neurotransmitters dock at the active zone of the presynaptic membrane. Priming: Fully filled vesicles undergo priming, becoming competent for Ca^2+^ −triggered fusion-pore opening. Fusion: Synaptotagmin-1 (SYT1) facilitates the release of neurotransmitters from the vesicles into the synaptic cleft. Endocytosis: Vesicles recycle via clathrin/Ap2-mediated endocytosis, returning to the presynaptic membrane for reuse. Some vesicles may also recycle through endosomes. Snap25, Vamp, clathrin, and Ap2 are among the key proteins involved in the synaptic vesicle cycle pathway, whose abundance has been observed to increase after lead exposure. This suggests that synaptic vesicle cycle signaling potentially plays a critical role in lead-induced ototoxicity.

The Shiny GO enrichment analysis of proteins whose abundance was increased after lead exposure indicated that BPs such as sodium ion export across the plasma membrane, clathrin-dependent endocytosis, and synaptic vesicle endocytosis were significantly enriched (Figures 7A,B). The KEGG pathways enrichment analysis revealed that the synaptic vesicle cycle is a highly enriched KEGG pathway (Figures 7C,D), and included proteins encoded by genes such as *Snap25, Vamp2*, *Slc1a2* that are associated with the vesicle-mediated transport. The network analysis of significantly enriched KEGG pathways indicated that the synaptic vesicle cycle pathway did not interact with the other enriched KEGG pathways (Figure 7C). The protein–protein-interaction (PPI) analysis of differentially expressed proteins in the synaptic vesicle cycle pathway, identified 22 nodes (proteins) and 39 edges (lines connecting nodes), with a PPI-enrichment value of <1.0 × 10^−16^ (Figure 7D). The illustration of the synaptic vesicle cycle KEGG pathway highlights the differentially expressed proteins (red) that play a critical role in this signaling pathway (Figure 8).

Additionally, bioinformatics analysis of proteins whose abundance was decreased after lead exposure indicated that BPs such as NADH regeneration, canonical glycolysis, and glucose catabolic process to pyruvate were significantly enriched (Supplementary Figures S1A,B). The KEGG pathways enrichment analysis revealed that 2-Oxocarboxylic acid metabolism, pyruvate metabolism and nitrogen metabolism pathways were highly enriched (Supplementary Figures S1C,D). The network analysis of significantly enriched KEGG pathways indicated that the interaction between these pathways was limited (Supplementary Figures S1C. The protein–protein-interaction (PPI) analysis of differentially abundant proteins in the 2-Oxocarboxylic acid metabolism pathway, identified 8 nodes and 14 edges, with a PPI-enrichment value of <1.0 × 10^−16^ (Supplementary Figure S1D).

## 4 Discussion

Environmental exposure to ototoxicants is partly responsible for inducing disabling hearing loss, which is estimated to affect 1 in every 10 people by 2050 (WHO, 2023b). Heavy metal lead, which is ubiquitous, affects auditory function after high-level exposures. Although the ototoxic effects of lead have been well documented, the underlying mechanisms are yet to be fully understood. This study provided evidence that outer hair cells are not the primary targets in lead-induced hearing loss. The immunostaining of the cochlear ribbon synapses indicated that the basal turn is more susceptible to lead-induced ototoxicity (Rosati et al., 2022). Furthermore, the mass spectrometry analyses identified critical synaptosomal proteins whose abundance is significantly altered by lead exposure while the bioinformatics analyses identified signaling pathways that are potentially associated with lead-induced cochlear synaptopathy.

Exposure of mice to lead in water for 28 days significantly increased their blood lead levels, which served as an indicator of the body burden. Consistent with previous studies (Jamesdaniel et al., 2018; Hu et al., 2019) lead exposure induced hearing loss in mice. Moreover, the lead-induced shift in hearing thresholds was similar in male and female mice suggesting that both sexes are susceptible to lead-induced ototoxicity. However, the otoacoustic emissions, even at higher frequencies, indicated that the activity of OHCs, which amplify and fine-tune sound signals within the cochlea (Goutman et al., 2015; Ashmore, 2019), were not affected by this lead exposure, which is consistent with the absence of auditory deficits after lead exposure observed in some studies (Carlson and Neitzel, 2018). Moreover, immunostaining of OHCs, which are highly susceptible to damage from noise and other ototoxic drugs, indicated that this lead exposure did not induce hair cell loss, even in the basal turn of the cochlea. These results indicate that lead exposure at concentrations that do not affect OHCs, can induce hearing impairment in mice and suggest that cochlear hair cell loss or death is not the primary target in lead-induced hearing impairment.

As lead exposure resulted in hearing loss without affecting the OHCs, other potential cochlear targets, such as the ribbon synapses, were examined. The ribbon synapses are crucial in transmitting signals from the IHCs to the auditory neurons and serve as the primary excitatory afferent synapses within the auditory pathway (Becker et al., 2018; Gao et al., 2020). The precise release of neurotransmitters at these synapses governs the firing pattern of the auditory nerve, which determines sound intensity and timing (Fuchs, 2005; Johnson et al., 2019). Therefore, any disruption in these ribbon synapses may contribute to the development of cochlear synaptopathy (Wei et al., 2020). The ABR wave I amplitudes are considered as an indicator of ribbon-synapse functionality (Yuan et al., 2020). A noticeable decrease in the amplitude of ABR wave-I was observed in lead-exposed animals compared to the control group. This suggested a reduction in the number of neurons responding to sound stimuli leading to cochlear synaptopathy. Moreover, consistent with previous studies (Rosati et al., 2022), lead exposure significantly decreased the expression of CtBP2 and GluR2, which are pre-and post-synaptic protein markers in IHC synapses, particularly in the basal turn of the Organ of Corti. The number of paired synapses was also significantly decreased in the basal turn after lead exposure suggesting lead-induced ribbon synaptic disruption and cochlear synaptopathy. As the decreases in both the ABR wave-I amplitude and the number of paired ribbon synapses were more pronounced in the basal region of the cochlea, these findings suggest that the basal region of the cochlea is more susceptible to lead-induced toxicity. Our data showed that the hair cells are intact, while there are significant differences in synaptic ribbon counts and a significant decrease in the number of paired synapses. Though we did not assess SGN counts, in a previous study, we reported lead-induced nitrative stress in SGNs (Rosati et al., 2022). Others have reported that lead exposure results in degenerative changes in SGNs (Hu et al., 2019), demyelination of neurons, and neurotoxic effects on both central and peripheral nerves (Seppäläinen and Hernberg, 1980; Bilińska et al., 2005; Zheng et al., 2011). Therefore, we assume that the ABR threshold shift is due to potential damage to the ribbon synapses, SGNs, and the cochlear nerves.

The connection between ribbon synapse disruption and hearing loss is well-established (Liu et al., 2016; Feng et al., 2020; Liu et al., 2022). A prior study demonstrated that lead exposure decreases the expression levels of presynaptic marker CtBP2 and postsynaptic marker GluR2, and disrupts the ribbon synapses in the cochlea (Rosati et al., 2022). In the current study, the proteomics changes underlying lead-induced disruption of ribbon synapses were analyzed to help identify crucial targets and signaling pathways associated with lead-induced cochlear synaptopathy. In addition, bioinformatics analysis of the cochlear synaptosomal proteins whose abundance was significantly altered by lead exposure was performed to identify the BPs and signaling pathways that are associated with lead-induced cochlear synaptopathy. As the extracted synaptic protein fraction is likely to contain both afferent and efferent synaptic material along with other possible contaminants, the observations of this study are relevant to the hair cell synapses and, to some extent, to efferent synapses.

Analysis of proteins whose abundance was increased after lead exposure indicated the enrichment of many BPs, including sodium ion export across plasma membrane, clathrin-dependent endocytosis, and synaptic vesicle endocytosis. These BPs play a key role in not only hair cell function, but also in vesicle recycling at presynaptic terminals, and the transport/release of neurotransmitters crucial for auditory neurotransmission. Lead-induced changes in these processes can lead to hearing impairment (Jin et al., 2009; Trune, 2010; Grabner and Moser, 2018; Wei et al., 2020). Analysis of associated KEGG pathways indicated that the synaptic vesicle cycle pathway is highly enriched (fold enrichment of 17.6) and included 22 proteins. This pathway plays a crucial role in the cochlear ribbon synapses and potential changes in the abundance of proteins that regulate this signaling can affect the transmission of auditory signals. Analysis of proteins whose abundance was decreased after lead exposure indicated the enrichment of many biological processes, including NADH regeneration, canonical glycolysis and glucose catabolic process to pyruvate. NAD^+^ metabolism and glucose hypometabolism are implicated in age-related hearing loss (Kim et al., 2014; Zainul Abidin et al., 2021). Moreover, NAD^+^ metabolism is also associated with protection against noise-induced hearing loss (Brown et al., 2014). NADH treatment rescued mitochondrial dysfunction, reduced cell apoptosis, and prevented hearing loss (Qiu et al., 2024). Factors that affect these processes can compromise cochlear function, and therefore, lead-induced changes in the synaptic proteins associated with these processes can contribute to hearing impairment. Analysis of associated KEGG pathways indicated that the 2-Oxocarboxylic acid metabolism pathway is highly enriched (fold enrichment of 22.2), although the potential link between the 8 proteins included in this pathway and hearing function is not known.

Neurotransmission through the chemical synapses relies on the synaptic vesicle cycle machinery, particularly on a continuous supply of receptors and proteins required for synaptic signaling (Watson et al., 2023). The synaptic vesicles play a vital role in neurotransmitter uptake, storage, and release during synaptic transmission (Volknandt, 1995). A substantial and rapidly releasable pool of synaptic vesicles is important for efficiently and accurately transmitting auditory information (Wei et al., 2020). So, lead-induced disruption of the synaptic proteins can significantly affect the transmission of signals. Among the cochlear synaptic proteins whose abundance was increased after lead exposure and are part of the synaptic vesicle cycle pathway, proteins encoded by genes such as *Ap2a1, Ap2m1, Slc17a6, Nsf, Slc1a2, Slc1a3, Snap25, Stxbp1, Vamp2, Ap2s1, Slc6a11, Stx1b, Ap2b1,* have been previously reported to be associated with auditory function. Specifically, *Ap2a1*, *Ap2m1*, *Ap2s1*, and *Ap2b1* are implicated in clathrin-mediated endocytosis (Di Rubbo et al., 2013; Lacruz et al., 2013) and IHC-specific deletion of *Ap2m1* induces profound hearing impairment in mice (Jung et al., 2015). *Slc17a6*, *Slc1a2*, *Slc1a3*, and *Slc6a11* can potentially influence synaptic transmission in the cochlear nucleus (CN) region of auditory system because *Slc17a6* and *Slc1a3* are upregulated in noise-induced and age-related hearing loss (Tadros et al., 2007; Manohar et al., 2016) and *Slc1a2* has been reported to play a crucial role in noise-induced cochlear synaptopathy by regulating glutamate clearance (Lavinsky et al., 2018). Moreover, *Nsf* is necessary for maintaining synaptic contacts between hair cells and afferent neurons (Mo and Nicolson, 2011) while SNARE proteins, including *Snap25*, *Stxbp1*, *Stx1b*, and *Vamp2*, mediate membrane fusion during vesicle trafficking and neurotransmitter release, which are crucial for normal exocytosis in neurons (Sollner et al., 1993; Salpietro et al., 2019; Cali et al., 2022). *Snap25* is essential for normal hearing function, ensuring IHC exocytosis, and ribbon synapse maintenance because mice with *Snap25* inactivation after hearing onset experience a significant level of hearing loss due to defective exocytosis, ribbon degeneration, and IHC loss (Calvet et al., 2022). *Vamp2* has been reported to play a role in the cochlear neuron development in nonhuman primates (Hosoya et al., 2021). Further, many of these proteins are linked to vesicle-mediated transport, fusion, clathrin coating assembly, and synaptic transmission, suggesting that the lead-induced changes in their abundance can influence neurotransmission across cochlear ribbon synapses. As the changes in these proteins can contribute to the lead-induced decrease in the ABR wave-I amplitudes, these proteins are likely to be crucial targets of lead-induced cochlear synaptopathy. Although we did not verify the changes in the abundance of proteins by alternate methods in this study, we have validated some of the changes detected by mass spectrometry analysis by using western blots in our recent study (Shahab et al., 2024). As the changes in the synaptosomal proteins may also reflect the general malfunction of protein expression regulation, further studies are needed to gain mechanistic insights into how these changes regulate lead-induced cochlear synaptopathy.

Collectively, the findings of this study demonstrate that lead-induced ototoxicity primarily targets cochlear synaptic function, particularly in the basal turn of the cochlea, even though other causes, such as damage to the spiral ganglions and demyelination of the cochlear nerve can also lead to reduced hearing sensitivity. The lead-induced cochlear synaptopathy is associated with changes in the abundance of many synaptosomal proteins, and the increase in the abundance of proteins encoded by genes such as *Snap25 and Vamp2 has* been associated with hearing loss in other studies. Conversely, the changes in the abundance of many other proteins, including those encoded by genes such as *Aco1, Bcat2* lack a direct link to hearing loss. While their involvement in cochlear stress response is unexplored, our findings pave the way for future research, to shed light on their involvement in the molecular mechanisms underlying lead-induced cochlear damage.

## Data availability statement

The datasets presented in this study can be found in online repositories. The mass spectrometry proteomics data have been deposited to the ProteomeXchange Consortium via the PRIDE partner repository with the dataset identifier PXD050415.

## Ethics statement

The animal study was approved by Institutional Animal Care and Use Committee, Wayne State University. The study was conducted in accordance with the local legislation and institutional requirements.

## Author contributions

PB: Data curation, Formal analysis, Investigation, Methodology, Writing – original draft. SM: Formal analysis, Resources, Visualization, Writing – original draft. ND-R: Investigation, Writing – original draft. RR: Formal analysis, Investigation, Methodology, Visualization, Writing – original draft. PS: Resources, Supervision, Writing – review & editing. SJ: Conceptualization, Funding acquisition, Project administration, Resources, Supervision, Writing – review & editing.

## Glossary

ABR: auditory brainstem response
BP: biological process
CtBP2: C-terminal-binding protein-2
DPOAE: distortion product otoacoustic emission
DTT: DL-Dithiothreitol
EDTA: ethylenediaminetetraacetic acid disodium salt
FA: formic acid
GluR2: Glutamate receptor 2
GO: Gene Ontology
IAA: Iodoacetamide
ICP-MS: inductively coupled plasma mass spectrometry
IHC: inner hair cells
KEGG: Kyoto Encyclopedia of Genes and Genomes
LC–MS/MS: Liquid Chromatography Tandem Mass Spectrometry
MeOH: methanol
NAD: Nicotinamide adenine dinucleotide
NADH: reduced nicotinamide adenine dinucleotide
OHCs: outer hair cells
PBS: phosphate buffered saline
PPI: protein–protein interaction
SNAP-25: Synaptosomal-Associated Protein 25
STRING: Search Tool for the Retrieval of Interacting Genes
TDT: Tucker Davis Technologies
TEAB: Triethylammonium bicarbonate buffer
