# Supplementary material for: Unraveling the molecular landscape of lead-induced cochlear synaptopathy: a quantitative proteomics analysis
Source: Front Cell Neurosci. 2024 Jul 22;18:1408208. doi: 10.3389/fncel.2024.1408208 (PMC11298392; doi:10.3389/fncel.2024.1408208)
Supplement: Supplementary file 1 [file Table_1.DOCX]

**Supplemental Data**

**Table S1:** KEGG pathways enriched by proteins whose abundance was increased by lead exposure.

| Pathway | N Genes | Pathway Genes | Fold Enrichment | Enrichment FDR | Gene name |
| --- | --- | --- | --- | --- | --- |
| [Synaptic vesicle cycle](http://www.genome.jp/kegg-bin/show_pathway?mmu04721) | 22 | 77 | 17.6 | 2.6E-19 | *Ap2a1, Ap2m1, Atp6v1a, Atp6v0d1, Atp6v1e1, Atp6v0a1, Dnm1, Slc17a6, Nsf, Slc1a2, Slc1a3, Snap25, Stxbp1, Vamp2, Ap2s1, Slc6a11, Stx1b, Atp6v1c1, Cltc, Ap2b1, Atp6v1d, Cltb* |
| [Proximal tubule bicarbonate reclamation](http://www.genome.jp/kegg-bin/show_pathway?mmu04964) | 6 | 22 | 16.8 | 1.2E-05 | *Atp1a1, Atp1b1, Atp1b2, Atp1b3, Atp1a3, Slc4a4* |
| [Aldosterone-regulated sodium reabsorption](http://www.genome.jp/kegg-bin/show_pathway?mmu04960) | 9 | 38 | 14.6 | 2.2E-07 | *Atp1a1, Atp1b1, Atp1b2, Atp1b3, Pik3r1, Atp1a3, Mapk1, Mapk3, Slc9a3r2* |
| [Collecting duct acid secretion](http://www.genome.jp/kegg-bin/show_pathway?mmu04966) | 6 | 27 | 13.7 | 3.6E-05 | *Atp6v1a, Atp6v0d1, Atp6v1e1, Atp6v0a1, Atp6v1c1, Atp6v1d* |
| [Endocrine and other factor-regulated calcium reabsorption](http://www.genome.jp/kegg-bin/show_pathway?mmu04961) | 13 | 61 | 13.1 | 1.2E-09 | *Ap2a1, Ap2m1, Atp1a1, Atp1b1, Atp1b2, Atp1b3, Atp2b2, Dnm1, Ap2s1, Atp1a3, Cltc, Ap2b1, Cltb* |
| [Oxidative phosphorylation](http://www.genome.jp/kegg-bin/show_pathway?mmu00190) | 18 | 133 | 8.3 | 7.6E-10 | *Cox6b1, Atp5j, Atp6v1a, Atp6v0d1, Atp6v1e1, Atp6v0a1, Cox7a2, mt-Atp8, mt-Co2, mt-Co3, Uqcrq, Atp5j2, Atp6v1c1, Ndufa7, Atp5e, Uqcrb, Ndufc2, Atp6v1d* |
| [Bacterial invasion of epithelial cells](http://www.genome.jp/kegg-bin/show_pathway?mmu05100) | 10 | 76 | 8.1 | 5.6E-06 | *Dnm1, Pik3r1, Septin8, Shc1, Septin3, Septin11, Cltc, Arpc5l, Cltb, Arhgap10* |
| [Cardiac muscle contraction](http://www.genome.jp/kegg-bin/show_pathway?mmu04260) | 11 | 87 | 7.8 | 3.3E-06 | *Atp1a1, Atp1b1, Atp1b2, Atp1b3, Cox7a2, mt-Co2, mt-Co3, Ryr2, Uqcrq, Atp1a3, Uqcrb* |
| [Gastric acid secretion](http://www.genome.jp/kegg-bin/show_pathway?mmu04971) | 9 | 75 | 7.4 | 3.3E-05 | *Atp1a1, Atp1b1, Atp1b2, Atp1b3, Camk2b, Gnai1, Itpr1, Atp1a3, Adcy1* |
| [Salivary secretion](http://www.genome.jp/kegg-bin/show_pathway?mmu04970) | 10 | 84 | 7.3 | 1.2E-05 | *Atp1a1, Atp1b1, Atp1b2, Atp1b3, Atp2b2, Itpr1, Slc12a2, Vamp2, Atp1a3, Adcy1* |
| [Gap junction](http://www.genome.jp/kegg-bin/show_pathway?mmu04540) | 10 | 86 | 7.2 | 1.4E-05 | *Csnk1d, Gja1, Gnai1, Itpr1, Tuba1a, Tubb4a, Tubb5, Mapk1, Mapk3, Adcy1* |
| [Insulin secretion](http://www.genome.jp/kegg-bin/show_pathway?mmu04911) | 10 | 86 | 7.2 | 1.4E-05 | *Atp1a1, Atp1b1, Atp1b2, Atp1b3, Camk2b, Ryr2, Snap25, Vamp2, Atp1a3, Adcy1* |
| [Glutamatergic synapse](http://www.genome.jp/kegg-bin/show_pathway?mmu04724) | 12 | 113 | 6.5 | 4.5E-06 | *Dlg4, Slc17a6, Gnai1, Gnao1, Itpr1, Ppp3ca, Ppp3cb, Slc1a2, Slc1a3, Mapk1, Mapk3, Adcy1* |
| [Pancreatic secretion](http://www.genome.jp/kegg-bin/show_pathway?mmu04972) | 12 | 113 | 6.5 | 4.5E-06 | *Atp1a1, Atp1b1, Atp1b2, Atp1b3, Atp2b2, Itpr1, Ryr2, Slc12a2, Atp1a3, Adcy1, Slc4a4, Atp1a2* |
| [Systemic lupus erythematosus](http://www.genome.jp/kegg-bin/show_pathway?mmu05322) | 14 | 142 | 6.1 | 2.0E-06 | *H4c17, Ctsg, H3f3b, Snrpd1, Macroh2a1, H3c3, H2bc8, H2bc9, H2bc14, H2bc18, Macroh2a2, H2az1, H2ac21, H2az2* |
| [Aldosterone synthesis and secretion](http://www.genome.jp/kegg-bin/show_pathway?mmu04925) | 10 | 102 | 6 | 5.6E-05 | *Atp1a1, Atp1b1, Atp1b2, Atp1b3, Atp2b2, Camk2b, Camk4, Itpr1, Atp1a3, Adcy1* |
| [Alcoholism](http://www.genome.jp/kegg-bin/show_pathway?mmu05034) | 18 | 199 | 5.6 | 1.5E-07 | *H4c17, Camk4, Gnai1, Gnao1, H3f3b, Shc1, Mapk1, Mapk3, Macroh2a1, H3c3, H2bc8, H2bc9, H2bc14, H2bc18, Macroh2a2, H2az1, H2ac21, H2az2* |
| [Adrenergic signaling in cardiomyocytes](http://www.genome.jp/kegg-bin/show_pathway?mmu04261) | 13 | 149 | 5.4 | 1.2E-05 | *Atp1a1, Atp1b1, Atp1b2, Atp1b3, Atp2b2, Camk2b, Gnai1, Ryr2, Atp1a3, Mapk1, Mapk3, Ppp2r5c, Adcy1* |
| [Prion disease](http://www.genome.jp/kegg-bin/show_pathway?mmu05020) | 22 | 266 | 5.1 | 2.2E-08 | *Cox6b1, Atp5j, Cox7a2, Itpr1, mt-Atp8, mt-Co2, mt-Co3, Ncam1, Pik3r1, Ppp3ca, Ppp3cb, Ryr2, Tuba1a, Tubb4a, Tubb5, Uqcrq, Mapk1, Mapk3, Ndufa7, Atp5e, Uqcrb, Ndufc2* |
| [Neutrophil extracellular trap formation](http://www.genome.jp/kegg-bin/show_pathway?mmu04613) | 16 | 201 | 4.9 | 3.7E-06 | *H4c17, Ctsg, H3f3b, Pik3r1, Mapk1, Mapk3, Macroh2a1, H3c3, H2bc8, H2bc9, H2bc14, H2bc18, Macroh2a2, H2az1, H2ac21, H2az2* |
| [CAMP signaling pathway](http://www.genome.jp/kegg-bin/show_pathway?mmu04024) | 17 | 219 | 4.8 | 2.6E-06 | *Atp1a1, Atp1b1, Atp1b2, Atp1b3, Atp2b2, Camk2b, Camk4, Gnai1, Pak1, Pik3r1, Ryr2, Atp1a3, Mapk1, Mapk3, Adcy1, Fxyd1, Atp1a2* |
| [Thermogenesis](http://www.genome.jp/kegg-bin/show_pathway?mmu04714) | 17 | 228 | 4.6 | 3.7E-06 | *Cox6b1, Atp5j, Cox7a2, Cpt1a, mt-Atp8, mt-Co2, mt-Co3, Slc25a29, Acsl6, Tsc2, Uqcrq, Adcy1, Atp5j2, Ndufa7, Atp5e, Uqcrb, Ndufc2* |
| [Parkinson disease](http://www.genome.jp/kegg-bin/show_pathway?mmu05012) | 19 | 262 | 4.5 | 1.7E-06 | *Cox6b1, Atp5j, Camk2b, Cox7a2, Gnai1, Itpr1, mt-Atp8, mt-Co2, mt-Co3, Septin5, Tuba1a, Tubb4a, Tubb5, Uqcrq, Ndufa7, Atp5e, Uqcrb, Ndufc2, Rps27a* |
| [Diabetic cardiomyopathy](http://www.genome.jp/kegg-bin/show_pathway?mmu05415) | 14 | 204 | 4.2 | 5.7E-05 | *Cox6b1, Atp5j, Camk2b, Cox7a2, mt-Atp8, mt-Co2, mt-Co3, Pik3r1, Ryr2, Uqcrq, Ndufa7, Atp5e, Uqcrb, Ndufc2* |
| [Amyotrophic lateral sclerosis](http://www.genome.jp/kegg-bin/show_pathway?mmu05014) | 25 | 365 | 4.2 | 6.2E-08 | *Cox6b1, Atp5j, Cox7a2, Matr3, mt-Atp8, mt-Co2, mt-Co3, Nefl, Nefm, Pfn2, Ppp3ca, Ppp3cb, Prph, Slc1a2, Tuba1a, Tubb4a, Tubb5, Uqcrq, Srsf7, Nefh, Ndufa7, Atp5e, Map1lc3b, Uqcrb, Ndufc2* |
| [Chemical carcinogenesis-reactive oxygen species](http://www.genome.jp/kegg-bin/show_pathway?mmu05208) | 15 | 220 | 4.2 | 3.3E-05 | *Cox6b1, Atp5j, Cox7a2, Gstt1, mt-Atp8, mt-Co2, mt-Co3, Pik3r1, Uqcrq, Mapk1, Mapk3, Ndufa7, Atp5e, Uqcrb, Ndufc2* |
| [Pathways of neurodegeneration-multiple diseases](http://www.genome.jp/kegg-bin/show_pathway?mmu05022) | 30 | 469 | 3.9 | 1.0E-08 | *Cox6b1, Atp5j, Camk2b, Cox7a2, Dlg4, Itpr1, mt-Atp8, mt-Co2, mt-Co3, Nefl, Nefm, Septin5, Ppp3ca, Ppp3cb, Prph, Ryr2, Tuba1a, Tubb4a, Tubb5, Uqcrq, Mapk1,*  *Mapk3, Csnk1e, Nefh, Ndufa7, Atp5e, Map1lc3b, Uqcrb, Ndufc2, Rps27a* |
| [Alzheimer disease](http://www.genome.jp/kegg-bin/show_pathway?mmu05010) | 22 | 380 | 3.6 | 4.5E-06 | *Cox6b1, Atp5j, Cox7a2, Itpr1, mt-Atp8, mt-Co2, mt-Co3, Pik3r1, Ppp3ca, Ppp3cb, Tuba1a, Tubb4a, Tubb5, Uqcrq, Mapk1, Mapk3, Csnk1e, Ncstn, Ndufa7, Atp5e, Uqcrb, Ndufc2* |

**Table S2:** KEGG pathways enriched by proteins whose abundance was decreased by lead exposure.

| Pathway | nGenes | Pathway Genes | Fold Enrichment | Enrichment FDR | Gene name |
| --- | --- | --- | --- | --- | --- |
| [2-Oxocarboxylic acid metabolism](http://www.genome.jp/kegg-bin/show_pathway?mmu01210) | 8 | 20 | 22.2 | 3.4E-08 | *Aco1, Bcat2, Cs, Got2, Idh1, Idh3g, Idh2, Gpt* |
| [Pyruvate metabolism](http://www.genome.jp/kegg-bin/show_pathway?mmu00620) | 13 | 44 | 16.4 | 2.3E-11 | *Glo1, Acat2, Aldh7a1, Adh5, Aldh2, Fh1, Hagh, Ldha, Ldhb, Me1, Mdh1, Pkm, Acyp2* |
| [Nitrogen metabolism](http://www.genome.jp/kegg-bin/show_pathway?mmu00910) | 5 | 17 | 16.3 | 1.2E-04 | *Car1, Car2, Car3, Glud1, Car13* |
| [Valine leucine and isoleucine degradation](http://www.genome.jp/kegg-bin/show_pathway?mmu00280) | 16 | 56 | 15.9 | 1.0E-13 | *Acat2, Aldh7a1, Pcca, Acadm, Acaa1a, Acads, Aldh2, Bcat2, Bckdha, Hibch, Acaa2, Ivd, Hibadh, Pccb, Oxct1, Mccc1* |
| [Carbon metabolism](http://www.genome.jp/kegg-bin/show_pathway?mmu01200) | 33 | 120 | 15.3 | 8.1E-28 | *Pgd, Acat2, Pcca, Acads, Aco1, Adh5, Aldoa, Cat, Cs, Eno2, Eno3, Esd, Fh1, Gapdh, Glud1, Got2, Gpi1, Idh1, Idh3g, Me1, Mdh1, Pgam1, Pgk1, Pkm, Taldo1, Tkt, Tpi1, Hibch, Idh2, Eno1b, Pgam2, Pccb, Gpt* |
| [Glycolysis/Gluconeogenesis](http://www.genome.jp/kegg-bin/show_pathway?mmu00010) | 18 | 66 | 15.1 | 5.4E-15 | *Aldh7a1, Adh5, Aldh2, Aldoa, Bpgm, Eno2, Eno3, Gapdh, Gpi1, Ldha, Ldhb, Pgam1, Pgk1, Pkm, Tpi1, Eno1b, Pgam2, Pgm1* |
| [Biosynthesis of amino acids](http://www.genome.jp/kegg-bin/show_pathway?mmu01230) | 21 | 78 | 14.9 | 2.5E-17 | *Aco1, Aldoa, Bcat2, Cs, Eno2, Eno3, Gapdh, Got2, Idh1, Idh3g, Pgam1, Pgk1, Pkm, Taldo1, Tkt, Tpi1, Mat2a, Idh2, Eno1b, Pgam2, Gpt* |
| [Glyoxylate and dicarboxylate metabolism](http://www.genome.jp/kegg-bin/show_pathway?mmu00630) | 7 | 31 | 12.5 | 1.6E-05 | *Acat2, Pcca, Aco1, Cat, Cs, Mdh1, Pccb* |
| [Propanoate metabolism](http://www.genome.jp/kegg-bin/show_pathway?mmu00640) | 7 | 31 | 12.5 | 1.6E-05 | *Pcca, Acads, Bckdha, Ldha, Ldhb, Hibch, Pccb* |
| [Citrate cycle (TCA cycle)](http://www.genome.jp/kegg-bin/show_pathway?mmu00020) | 7 | 32 | 12.1 | 1.9E-05 | *Aco1, Cs, Fh1, Idh1, Idh3g, Mdh1, Idh2* |
| [Pentose phosphate pathway](http://www.genome.jp/kegg-bin/show_pathway?mmu00030) | 6 | 33 | 10.1 | 2.9E-04 | *Pgd, Aldoa, Gpi1, Taldo1, Tkt, Pgm1* |
| [Fatty acid degradation](http://www.genome.jp/kegg-bin/show_pathway?mmu00071) | 9 | 52 | 9.6 | 6.6E-06 | *Acat2, Aldh7a1, Acadl, Acadm, Acaa1a, Acads, Adh5, Aldh2, Acaa2* |
| [Alanine aspartate and glutamate metabolism](http://www.genome.jp/kegg-bin/show_pathway?mmu00250) | 6 | 38 | 8.8 | 5.6E-04 | *Adsl, Adssl1, Adss, Glud1, Got2, Gpt* |
| [Glutathione metabolism](http://www.genome.jp/kegg-bin/show_pathway?mmu00480) | 11 | 72 | 8.5 | 1.6E-06 | *Pgd, Prdx6, Gpx1, Gpx3, Gstm2, Gstp1, Idh1, Srm, Idh2, Mgst1, Oplah* |
| [Complement and coagulation cascades](http://www.genome.jp/kegg-bin/show_pathway?mmu04610) | 14 | 92 | 8.4 | 3.4E-08 | *C8b, Serping1, C3, F10, F3, F9, Cfb, Serpinf2, Serpina1a, Serpina1b, Serpina1c, Serpina1d, Vtn, F13a1* |
| [Cysteine and methionine metabolism](http://www.genome.jp/kegg-bin/show_pathway?mmu00270) | 8 | 53 | 8.4 | 6.0E-05 | *Ahcyl, Bcat2, Got2, Ldha, Ldhb, Mdh1, Srm, Mat2a* |
| [Nucleotide metabolism](http://www.genome.jp/kegg-bin/show_pathway?mmu01232) | 12 | 84 | 7.9 | 9.8E-07 | *Adsl, Adssl1, Adss, Ampd3, Aprt, Entpd1, Nme1, Nme2, Pnp, Xdh, Ampd1, Ntpcr* |
| [Proteasome](http://www.genome.jp/kegg-bin/show_pathway?mmu03050) | 6 | 47 | 7.1 | 1.7E-03 | *Psmb1, Psmb6, Psmb7, Psma1, Psma6, Psma7* |
| [ECM-receptor interaction](http://www.genome.jp/kegg-bin/show_pathway?mmu04512) | 11 | 88 | 6.9 | 9.4E-06 | *Npnt, Col2a1, Col4a2, Col9a2, Col1a1, Col1a2, Comp, Dmp1, Ibsp, Spp1, Vtn* |
| [HIF-1 signaling pathway](http://www.genome.jp/kegg-bin/show_pathway?mmu04066) | 13 | 113 | 6.4 | 3.0E-06 | *Aldoa, Eno2, Eno3, Gapdh, Igf1, Ldha, Ldhb, Pgk1, Trf, Vegfa, Eno1b, Elob, Eloc* |
| [Fatty acid metabolism](http://www.genome.jp/kegg-bin/show_pathway?mmu01212) | 7 | 62 | 6.3 | 1.2E-03 | *Acat2, Acadl, Acadm, Acaa1a, Acads, Hacd1, Acaa2* |
| [Purine metabolism](http://www.genome.jp/kegg-bin/show_pathway?mmu00230) | 14 | 134 | 5.8 | 3.1E-06 | *Adsl, Adssl1, Adss, Ampd3, Aprt, Entpd1, Nme1, Nme2, Pnp, Xdh, Ampd1, Pfas, Ntpcr, Pgm1* |
| [Central carbon metabolism in cancer](http://www.genome.jp/kegg-bin/show_pathway?mmu05230) | 7 | 69 | 5.6 | 2.1E-03 | *Idh1, Ldha, Ldhb, Pgam1, Pkm, Idh2, Pgam2* |
| [PPAR signaling pathway](http://www.genome.jp/kegg-bin/show_pathway?mmu03320) | 9 | 89 | 5.6 | 3.7E-04 | *Acadl, Acadm, Acaa1a, Adipoq, Fabp4, Apoa2, Apoc3, Fabp3, Me1* |
| [Hypertrophic cardiomyopathy](http://www.genome.jp/kegg-bin/show_pathway?mmu05410) | 9 | 91 | 5.5 | 4.3E-04 | *Atp2a1, Cacna1s, Myh7, Igf1, Myl2, Tpm1, Ttn, Tpm4, Tpm3* |
| [Dilated cardiomyopathy](http://www.genome.jp/kegg-bin/show_pathway?mmu05414) | 9 | 94 | 5.3 | 5.3E-04 | *Atp2a1, Cacna1s, Myh7, Igf1, Myl2, Tpm1, Ttn, Tpm4, Tpm3* |
| [Biosynthesis of cofactors](http://www.genome.jp/kegg-bin/show_pathway?mmu01240) | 14 | 152 | 5.1 | 1.2E-05 | *Adsl, Adssl1, Adss, Aldh2, Bcat2, Cpox, Alad, Nme1, Nme2, Pdxk, Uros, Mat2a, Coq5, Pmm2* |
| [Cardiac muscle contraction](http://www.genome.jp/kegg-bin/show_pathway?mmu04260) | 8 | 87 | 5.1 | 1.6E-03 | *Atp2a1, Cacna1s, Cox6a2, Myh7, Myl2, Tpm1, Tpm4, Tpm3* |
| [Metabolic pathways](http://www.genome.jp/kegg-bin/show_pathway?mmu01100) | 112 | 1611 | 3.9 | 1.2E-34 | *Glo1, Pgd, Acat2, Aldh7a1, Pcca, Acadl, Acadm, Acaa1a, Acads, Aco1, Acp1, Adh5, Adsl, Adssl1, Adss, Ahcyl, Aldh1a1, Aldh2, Aldoa, Ampd3, Prdx6, Aprt, Bcat2, Bckdha, Smyd1, Bpgm, Car1, Car2, Car3, Cat, Entpd1, Ckm, Cox6a2, Cpox, Cs, Cyp2a4, Cyp2a5, Eno2, Eno3, Esd, Fah, Fh1, Gapdh, Hagh, Glud1, Got2, Gpi1, Gpx1, Gpx3, Gstm2, Gstp1, Gstz1, Hal, Idh1, Idh3g, Ldha, Ldhb, Lta4h, Alad, Mif, Me1, Mdh1, Nme1, Nme2, Oat, Pgam1, Pgk1, Pkm, Pnp, Pygm, Srm, Chpt1, Taldo1, Pomgnt2, Pdxk, Inmt, Gmds, Tkt, Tpi1, Uros, Xdh, Hibch, Ampd1, Mat2a, Pfas, Idh2, Aass, Hacd1, Eno1b, Coq5, Acaa2, Pmm2, Pgam2, Ivd, Mgst1, Hibadh, Nampt, Atp6v1g1, Ntpcr, Pccb, Oxct1, Lipf, Cmbl, Ndufab1, Car13, Mccc1, Pgm1, Oplah, Acyp2, Grhpr, Gpt, Nans* |
| [Focal adhesion](http://www.genome.jp/kegg-bin/show_pathway?mmu04510) | 12 | 200 | 3.3 | 2.4E-03 | *Col2a1, Col4a2, Col9a2, Col1a1, Col1a2, Comp, Ibsp, Igf1, Myl2, Spp1, Vegfa, Vtn* |


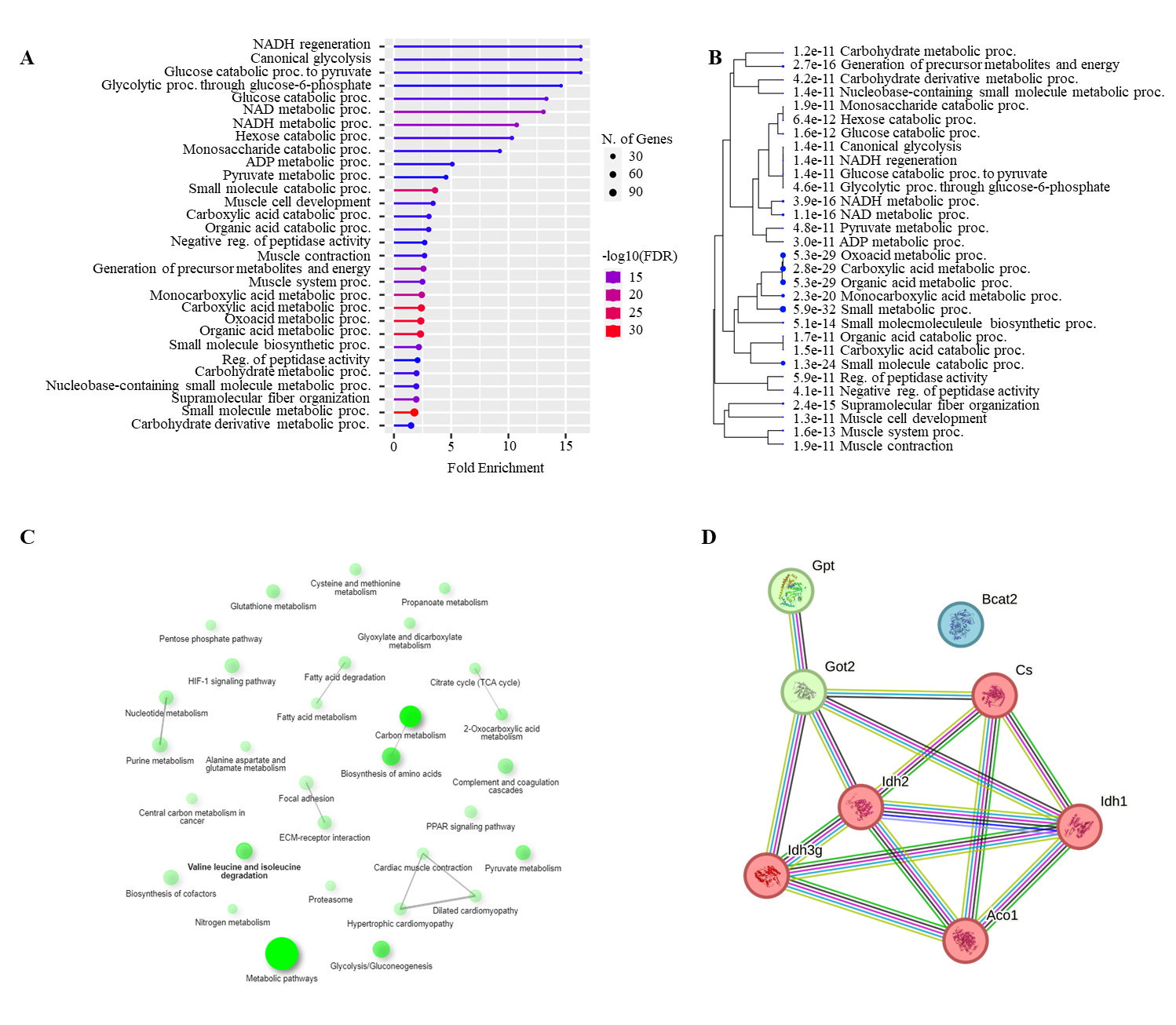


**Figure S1:**  Bioinformatics analysis of cochlear synaptosomal proteins whose abundance was decreased after lead exposure. **(A)** The GO analysis using the Shiny GO platform revealed the BPs enriched in the data set. The dot plot illustrates the fold enrichment, FDR, and the total number of proteins associated with these BPs. **(B)** The hierarchical clustering tree illustrates the clustering of significantly enriched BPs (p-value cutoff = 0.05). **(C)** The network analysis of enriched KEGG pathways shows the number of proteins in the pathway (indicated by the size of the node) and the percentage of overlapping proteins (indicated by the thickness of the edges). **(D)** The STRING interaction analysis of proteins associated with the 2-Oxocarboxylic acid metabolism pathway illustrates the type of interaction with the highest confidence (0.900). Three main clusters, cluster 1 represented by red nodes, cluster 2 represented by green nodes and cluster 3 represented by light blue nodes were detected. GO, Gene Ontology; BPs, Biological processes; FDR, False discovery rate; STRING, Search Tool for the Retrieval of Interacting Genes.
